# Análisis de los determinantes próximos e impacto de la ocupación en la fertilidad de mujeres peruanas

**DOI:** 10.26633/RPSP.2017.18

**Published:** 2017-03-06

**Authors:** Yordanis Enríquez-Canto, Katherine Ortiz-Romaní, Yonathan Ortiz-Montalvo

**Affiliations:** 1 Universidad Católica Sedes Sapientiae Facultad de Ciencias de la Salud Lima Perú Universidad Católica Sedes Sapientiae, Facultad de Ciencias de la Salud, Lima, Perú.

**Keywords:** Fertilidad, maternidad, carga de trabajo, Perú, Fertility, parenting, workload, Peru

## Abstract

**Objetivo.:**

*Estimar la capacidad predictiva de los determinantes próximos y el trabajo en la disminución de la fertilidad en mujeres peruanas en edad fértil*.

**Métodos.:**

*Análisis secundario de la Encuesta Demográfica y de Salud Familiar (ENDES) de 2014 de Perú. La muestra fue de 20 396 mujeres entre 15 y 49 años de edad con actividad sexual en las cuatro semanas previas a la entrevista realizada. La variable dependiente fue el número total de hijos nacidos vivos y las variables independientes principales incluidas, trabajo, número ideal de hijos, máximo nivel educativo alcanzado y quintiles de riqueza. Se calcularon* odds ratios *(OR) para estimar la fuerza de las asociaciones entre los determinantes próximos y la ocupación y la fertilidad mediante modelos de regresión logística. Se estimó la bondad de ajuste de los modelos con la prueba de Hosmer-Lemeshow y su capacidad discriminante, con curvas ROC*.

**Resultados.:**

*La fertilidad deseada (2,5 hijos por mujer) fue más alta que la real* *(2,1). Los principales factores asociados con la reducción de la fertilidad fueron el nivel educativo superior (OR = 0,03; IC95%: 0,02*–*0,04), el número ideal de hijos entre 0 y 2 (OR = 0,13; IC95%: 0,11*–*0,15), y el trabajo dependiente (OR = 0,31; IC95%: 0,28*–*0,34). El área bajo la curva de los modelos fue 0,908 (IC95%: 0,898*–*0,917) y 0,91 (IC95%:0,891*–*0,928), respectivamente*.

**Conclusión.:**

*La acción de los determinantes directos (edad de inicio de las relaciones sexuales y del matrimonio) retrasa el comienzo de la maternidad, mientras que los determinantes indirectos (mayor nivel educativo y pertenencia al quintil superior de riqueza) suponen una mayor autonomía económica, que, a su vez, se asocia con niveles bajos de fertilidad. Asimismo, cuanto mayor es la dependencia en el trabajo, menor es la fertilidad de las mujeres encuestadas*.

## Abstract (en)

**Objective:**

To estimate the predictive capacity of proximate determinants and type of employment with respect to reduced fertility in Peruvian women of childbearing age.

**Methods:**

Secondary analysis of Peru’s Demographic and Family Health Survey (ENDES) for 2014. The sample consisted of 20 396 women aged 15 to 49 years who had been sexually active during the four-week period immediately before the interview. The dependent variable was the total number of live births, and the main independent variables were type of employment, ideal number of offspring, highest educational level attained, and wealth quintile. Odds ratios (OR) were used to estimate the strength of the associations between proximate determinants and type of employment on the one hand and fertility on the other in logistic regression models. The goodness of fit of the models was estimated using the Hosmer-Lemeshow test and its discriminatory capacity, based on receiver operating characteristic curves.

**Results:**

Desired fertility (2.5 children per woman) was higher than actual fertility (2.1). The factors most strongly associated with reduced fertility were higher educational attainment (OR = 0.03; 95% CI: 0.02-0.04); ideal number of offspring between 0 and 2 (OR = 0.13; 95% CI: 0.11-0.15); and dependent employment (OR = 0.31; 95% CI: 0.28-0.34). The models’ areas under the curve were 0.908 (95% CI: 0.898-0.917) and 0.91 (95% CI: 0.891-0.928), respectively.

**Conclusion:**

Older age at first sexual intercourse and older age when first married (direct determinants) delay childbearing, whereas higher educational level and belonging to the highest wealth quintile (indirect determinants) indicate greater economic autonomy, which, in turn, is associated with low fertility. Similarly, the more dependent the work, the lower the fertility of the women surveyed.

La disminución de la fertilidad que se observa en los países en desarrollo desde 1960 (1, 2) puede ser parcialmente explicada por la acción de los determinantes próximos de la fertilidad (1–4). Este proceso es complejo, pues existen diferencias individuales en las preferencias de fertilidad relacionadas con los deseos de autorrealización profesional y de desarrollo personal (5–7), así como con cambios en los ideales del tamaño de la familia (8, 9). La progresión de esta tendencia a la reducción está relacionada con un proceso de retraso de la maternidad caracterizado por un incremento de la media de edad de las madres al tener el primer hijo (1, 9). Sin embargo, la contribución de cada uno de los determinantes no se ha podido explicar completamente.

En Perú, los niveles de fertilidad siguen la tendencia mundial a la reducción. Este decrecimiento ha sido notable, ya que ha descendido del 4,3 hijos por mujer en 1986 a 2,5 en 2014 (10). A escala de la Región de las Américas, esta tendencia es heterogénea (2, 11). No obstante, una característica común en la Región es el aumento de la participación de la mujer en el mercado laboral y en la inversión en capital humano (1, 12), lo que repercute positivamente en el status y en la autonomía de las mujeres y aumenta el costo de oportunidad de la maternidad (2). En la medida en que ha aumentado la participación de la mujer en el mercado laboral retribuido, y con el aumento de los núcleos familiares mantenidos por dos fuentes de ingresos, han disminuido los niveles de fertilidad (12). Por otro lado, los países de la Región han implementado políticas, como la licencia de maternidad, para favorecer la reducción de la incompatibilidad entre el trabajo y la vida familiar. Sin embargo, estas garantías no son universalmente accesibles, dado que, por ejemplo, se ha notificado que 77,6% de las mujeres peruanas tienen un trabajo informal que les impide acceder a las licencias de maternidad (13). Debe resaltarse que en 2014 la población peruana económicamente activa ocupada fue de 15 796 885 personas, 43,8% de las cuales eran mujeres, que representan 49,9% del total de la población (30 814 175) (14).

Las decisiones sobre la maternidad son fundamentales para explicar el cambio de la estructura familiar y están mediadas por la norma social y por los valores, deseos e intenciones de las personas (15). En un creciente número de estudios se propone que la fertilidad deseada (el número ideal de hijos) afecta a la fertilidad real y estas investigaciones se focalizan en la discrepancia entre ambas (*fertility gap*) (16–19), que puede ser positiva debido a que la fertilidad deseada es mayor que la fertilidad real (19–22). Teniendo en cuenta estos hechos, se ha formulado la hipótesis según la cual en el grupo de mujeres peruanas con estudios superiores, que se han incorporado al mercado laboral, que pertenecen al quintil superior de riqueza y que tienen una valoración positiva de las familias pequeñas, los niveles de fertilidad son bajos.

En los estudios sobre la reducción de los niveles de fertilidad se han considerado diferentes factores (directos e indirectos) para explicar dicha disminución (12, 15, 23–27). Conocer los cambios en los comportamientos relacionados con la fertilidad y las variaciones en los niveles de fertilidad en los grupos de población de un país es fundamental para proponer políticas públicas de salud, ya que estas transformaciones se reflejan en el tamaño y en la estructura de la población (9, 28). En este sentido, es necesario seguir investigando los factores relacionados con la disminución de los niveles de fertilidad con el fin de mejorar tanto la comprensión del peso específico de cada uno de los determinantes, como la eficacia de las intervenciones en grupos poblacionales de riesgo. El objetivo de este estudio fue evaluar los determinantes próximos en la disminución de la fertilidad y el trabajo como factor predictor de la misma.

## MATERIALES Y MÉTODOS

En el presente estudio se empleó el modelo de Bongaarts para construir el marco conceptual de aproximación al problema y seleccionar y categorizar las variables del estudio (3, 4). Se utilizó la revisión de J. Stover (29), que emplea la proporción sexual activa de mujeres (con actividad sexual en los últimos 30 días) teniendo en cuenta el hecho de que la fertilidad no solo se produce dentro de la pareja, puesto que la misma no es un indicador fiable de la exposición a la actividad sexual en muchos países, sino fuera de ella.

Se realizó un análisis secundario de los datos de la Encuesta Demográfica y de Salud Familiar (ENDES) del 2014 (10). El muestreo de mujeres fue bietápico por conglomerados y estratificado por áreas y zonas de los departamentos del Perú. En la ENDES se entregaron individualmente cuestionarios a mujeres de 15 a 49 años de edad con el fin de recopilar información relativa a la dinámica demográfica y a su estado de salud y el de sus hijos menores de cinco años. La muestra del estudio fue de 20 396 mujeres en edad fértil y en ella se incluyeron solo las que fueron sexualmente activas en las cuatro semanas previas a la encuesta independientemente de su estado civil. De este modo, se acepta el supuesto de que la actividad sexual tiene mayor nivel predictivo que la unión en pareja. Las mujeres que nunca habían tenido relaciones sexuales se excluyeron del estudio.

El indicador específico de la fertilidad utilizado fue el total de hijos nacidos vivos (HNV). La variable dependiente fue el número total de HNV durante la edad fértil de la mujer hasta el momento de la entrevista y se calculó considerando a todos los hijos, incluidos los que fallecieron en los períodos perinatal y neonatal. La variable se originó a partir de las siguientes preguntas: ¿Ha tenido alguna hija o hijo nacido vivo?; ¿Cuántas hijas o hijos viven con usted?; ¿Cuántas hijas o hijos no están viviendo con usted?; ¿Cuántas hijas o hijos han muerto?; y se estratificó en las siguientes categorías: ningún hijo, de uno hasta tres hijos y de cuatro a más hijos. Las variables independientes fueron conceptualizadas teniendo en cuenta el modelo de Bongaarts, que incluye el análisis de dos grupos de factores, los directos y los indirectos (3, 4).

Por un lado, los factores directos fueron los siguientes. Estado civil, variable categórica: nunca se casó, unidas (casadas y convivientes) y otras (separadas, viudas y divorciadas). Uso actual de un método anticonceptivo*,* definida como el método que está usando la entrevistada o su pareja para evitar el embarazo, generada a partir de la pregunta ¿Qué están haciendo o usando para evitar quedar embarazada?, y definida como categórica (ningún método, tradicional y moderno). Exposición, definida como el período de infertilidad caracterizado por la ausencia del ciclo ovulación-menstruación, categorizada como sí o no, y generada a partir de la variable infecundabilidad postparto (con amenorrea y sin amenorrea). Mortalidad intrauterina y aborto espontáneo, variable categórica (sí y no). Edad en el primer matrimonio, variable categórica (10–18 años, 19–24 años y 25–46 años). Edad al nacimiento de su primer hijo, variable categórica (11–18 años, 19–24 años y 25–46 años), y Edad en la primera relación sexual, variable categórica (8–18 años, 19–24 años y 25–49 años).

Por otro lado, los indirectos fueron los siguientes. Edad de la mujer, variable categórica (15–19 años, 20–24 años, 25–29 años, 30–34 años, 35–39 años 40–44 años y 45–49 años). Residencia, variable categórica dicotómica (urbana y rural). Trabajo*,* definida como el tipo de ocupación laboral realizada en los últimos doce meses*,* y categórica (para un familiar, para alguien más e independiente). Ocupación laboral actual, definida como la realización de actividad laboral en la semana anterior a la encuesta, y categórica (sí, no). Tipo de paga por el trabajo, variable categórica (no le pagan, sólo dinero, dinero-especie y sólo especie). Máximo nivel educativo alcanzado, variable categórica (sin educación, primaria, secundaria y superior). Quintiles de riqueza, definidos en función de los activos o la riqueza en los hogares encuestados. En lugar de ingresos o consumos, el quintil depende de la disponibilidad de bienes y servicios y de las características de la vivienda, como, por ejemplo, el acceso a servicios básicos de agua potable, electricidad, la presencia de servicios higiénicos, y la disponibilidad de bienes de consumo duraderos en el hogar. También es una variable categórica (quintil superior, cuarto quintil, quintil intermedio, segundo quintil y quintil inferior). Número ideal de hijos, definida como el número ideal de hijos que la entrevistada desearía tener durante su vida*,* y considerada asimismo categórica (0–2, 3, 4, 5 o más hijos).

Para realizar el análisis estadístico, se construyeron tablas de contingencia utilizando el programa Stata SE 12 y se calcularon las medidas de medidas de tendencia central de los determinantes próximos y de la variable dependiente. La fuerza de la asociación entre el total de HNV y las variables independientes se estimó con la prueba de Chi-cuadrado. A partir de las tablas de contingencia, las variables con las cuales se encontraron diferencias estadísticamente significativas en el análisis bivariante usando como punto de corte un valor *P* < 0,20 se incluyeron en el modelo de regresión logística. Con este modelo se estimaron los odds ratios y sus intervalos de confianza de 95%. La bondad de ajuste de los modelos finales se estimó con la prueba de Hosmer-Lemeshow y su capacidad discriminante, con curvas ROC en las cuales se estimó el área bajo la curva y su intervalo de confianza de 95%.

La ENDES se basa en un tipo de muestreo complejo por estratificación, conglomerados y tasas de respuesta y, por este motivo, en todos los análisis se consideraron valores ponderados y no ponderados. En este estudio, así como en la encuesta ENDES 2014, se garantizó el anonimato de las encuestadas, para impedir que se identificara información personal sensible. Todas las mujeres encuestadas dieron su consentimiento informado verbalmente y el estudio fue aprobado por el Comité de Ética de la Universidad Católica Sedes Sapientiae de Lima.

## RESULTADOS

La media de la edad de las 20 396 mujeres entrevistadas de la muestra fue de 33 años, 65% tenía de uno a tres hijos y 69,0% vivía con su pareja. El método anticonceptivo más utilizado fue el moderno (45%) (cuadro 1). En los últimos 12 meses, 78% (15 914) trabajaba, 68% (13 848) de las cuales en la semana anterior a la encuesta, 51% para alguien (trabajo dependiente), y 81% recibió la retribución solo en dinero. El 47% de las mujeres que trabajaron para un familiar no recibió remuneración, y 9% del total no recibió remuneración alguna.

**CUADRO 1.** Descripción de los determinantes próximos de la fertilidad de las mujeres peruanas en edad fértil entrevistadas, Perú, 2014

Porcentaje			
No.	No ponderado	Ponderado		
Total de mujeres	20 396	
Total de hijos				
	Ninguno	3 222	15,8	18,1
	1–3	12 950	63,5	64,5	
	≥ 4 hijos	4 224	20,7	17,4	
Directos				
Edad en el primer matrimonio (años)				
	Media ± DE	(20,3 ± 4,9)	(20,8 ± 5,0)
	10–18	7 218	42,1	38,4
	19–24	6 923	40,3	41,7
	25–46	3 015	17,6	19,9
Edad al nacimiento de su primer hijo (años)				
	Media ± DE		(20,8 ± 4,5)	(21,3 ± 4,8)
	11–18	6 051	35,2	31,5
	19–24	8 050	46,9	47,1
	25–46	3 073	17,9	21,3
Edad en la primera relación sexual (años)				
	Media ± DE		(18,0 ± 3,6)	(18,3 ± 3,7)
	8–18	13 415	65,8	62,3
	19–24	5 825	28,6	31,1
	25–49	1 156	5,7	6,6
Estado civil				
	Nunca se casó	3 240	15,89	17,8
	En pareja	14 489	71,04	68,6
	Otro	2 667	13,08	13,6
Uso actual de método anticonceptivo				
	Ningún método	7 816	38,3	38,0
	Tradicional	3 648	17,9	17,5
	Moderno	8 932	43,8	44,5
Exposición				
	Sin amenorrea	18 764	92,0	92,6
	Con amenorrea	1 632	8,0	7,4
Mortalidad intrauterina y aborto espontáneo				
	No	16 130	79,1	78,6
	Sí	4 266	20,9	21,4
Indirectos				
Número ideal de hijos				
	≥ 5	1 006	4,9	4,8
	4	2 238	11,0	10,1
	3	4 380	21,5	22,0
	0–2	12 772	62,6	63,1
Edad de la mujer (años)				
	Media ± DE		(33,1 ± 9,1)	(33,2 ± 9,1)
	15–19	1 404	6,9	6,5
	20–24	2 911	14,3	14,4
	25–29	3 374	16,5	16,7
	30–34	3 484	17,1	16,9
	35–39	3 336	16,4	16,5
	40–44	3 071	15,1	15,4
	45–49	2 816	13,8	13,6
Residencia				
	Urbana	13 832	67,8	77,6
	Rural	6 564	32,2	22,4
Trabajo				
	Para un familiar	2 902	18,2	15,3
	Para alguien más	7 248	45,6	50,8
	Independiente	5 764	36,2	33,9
Tipo de paga por el trabajo				
	No le pagan	2 123	13,3	8,8
	Solo dinero	11 981	75,3	80,7
	Dinero y especie	1 465	9,2	8,7
	Solo especie	345	2,2	1,8
Máximo nivel educativo alcanzado				
	Sin educación	558	2,8	2,3
	Primaria	5 228	25,6	21,1
	Secundaria	8 566	42,0	43,3
	Superior	6 044	29,6	33,2
Quintiles de riqueza				
	Quintil inferior	4 778	23,4	16,2
	Segundo quintil	4 957	24,3	20,1
	Quintil intermedio	4 297	21,1	21,8
	Cuarto quintil	3 493	17,1	21,6
	Quintil superior	2 871	14,1	20,2

La media del número de hijos de las entrevistadas fue 2,09 mientras que la media del número ideal de hijos (fertilidad deseada) fue 2,47. Entre las mujeres que trabajaban, la media del número de hijos fue 2,10 y la media del número ideal de hijos, 2,46. Entre las que no han tenido hijos, 73% trabajó para alguien y 48% de las mujeres con cuatro hijos o más tuvo un trabajo independiente. De las mujeres con entre uno y tres hijos, el primero lo tuvieron entre los 19 y los 24 años de edad. Sin embargo, 53% de las que tenían cuatro o más hijos tuvo el primero cuando eran adolescentes. El resto de los determinantes se puede apreciar en el cuadro 2.

**CUADRO 2.** Perfil sociodemográfico y determinantes de las mujeres peruanas en edad fértil según los subgrupos de fertilidad, Perú, 2014

Determinantes	Subgrupos		
	Ningún hijo (%)	De 1 a 3 hijos (%)	≥ 4 hijos (%)
Edad de la mujer	20–24 (33)	30–34 (20)	45–49 (32)
Estado civil	Nunca se casó (72)	Unidas (77)	Unidas (86)
Edad en el primer matrimonio	19–24 (38)	19–24 (44)	10–18(58)
Edad al nacer su primer hijo	…	19–24 (48)	11–18 (53)
Edad en la primera relación sexual	8–18 (50)	8–18 (61)	8–18 (81)
Uso actual de método anticonceptivo	Ninguno (60)	Moderno (48)	Moderno (48)
Exposición	…	Sí (9)	Sí (9)
Embarazo terminado en perdida o aborto	Sí (10)	Sí (23)	Sí (25)
Residencia	Urbano (89)	Urbano (80)	Urbano (57)
Trabajo	Para alguien (73)	Para alguien (49)	Independiente (48)
Tipo de paga	Solo dinero (85)	Solo dinero (83)	Solo dinero (67)
Máximo nivel educativo	Superior (58)	Secundaria (48)	Primaria (52)
Quintiles de riqueza	Superior (31)	Intermedio (23)	Inferior (36)
Número ideal de hijos	0–2 (76)	0–2 (63)	0–2 (50)

***Fuente***: Elaboración propia a partir de los datos de la ENDES 2014.

En el cuadro 3 se muestran los resultados del análisis del modelo de regresión logística con todas las mujeres entrevistadas. Como se puede observar, el mayor riesgo de disminución de la fertilidad está asociado con las mujeres que tienen mayor nivel de educación, pertenecen a los quintiles superiores de riqueza y cuyo número ideal de hijos es menor de dos. La edad temprana en el primer matrimonio (10–18 años) se asocia con un aumento de la fertilidad respecto a las que tienen de 19 a 24, y en las de 25 y más años de edad el riesgo de pérdida de la fertilidad es mayor que en las de 19 a 24. El retraso en la iniciación sexual se asocia asimismo con una reducción de la fertilidad, a semejanza de lo que ocurre con las que trabajan para un familiar o para alguien más. Estos datos se comportan de modo similar en el subgrupo poblacional de mujeres que trabajaron en la última semana antes de la encuesta (cuadro 4). Las bondades de ajuste de los dos modelos fueron (*x* ^2^ = 5,463.3, gl = 8; *P* = 0,707) y (*x*^2^ = 7,061, gl = 8; *P* = 0,530). Sus capacidades discriminantes, evaluadas mediante el área bajo la curva (ABC), fueron 0,908 (IC95%: 0,898–0,917) y 0,91 (IC95%: 0,891–0,928), respectivamente.

## DISCUSIÓN

Los resultados muestran que las mujeres del estudio tienen en promedio 2,09 hijos y que la disminución de la fertilidad se asocia con mayor nivel educativo, una valoración positiva de las familias pequeñas (número ideal de hijos de cero a dos), el retraso en el inicio de las relaciones sexuales (después de los 24 años), el aplazamiento de la edad en el primer matrimonio (después de los 24 años) y una mayor autonomía: pertenencia al quintil superior, trabajo dependiente y tipo de retribución solo en dinero.

**CUADRO 3.** Predictores de la disminución de los niveles de fertilidad de todas las mujeres peruanas en edad fértil entrevistadas (n = 20 396), Perú, 2014

Determinantes	OR crudo^a^			
	OR	IC95%	P
Directos				
Edad en el primer matrimonio (años)				
	10–18	2,08	(1,89–2,29)	< 0,001
	19–24	1,00	Referencia	
	25–46	0,43	(0,39–0,49)	< 0,001
Edad en la primera relación sexual (años)				
	8–18	1,00	Referencia	
	19–24	0,46	(0,42–0,50)	< 0,001
	25–49	0,35	(0,31–0,41)	< 0,001
Edad al nacimiento del primer hijo (años)				
	11–18	1,00	Referencia	
	19–24	0,44	(0,43–0,49)	< 0,001
	25–46	0,07	(0,06–0,09)	< 0,001
Estado civil				
	Nunca se casó	1,00	Referencia	
	Con pareja	46,2	(40,4–52,9)	< 0,001
	Otro	34,6	(29,3–40,9)	< 0,001
Uso actual de método anticonceptivo				
	Ningún método	1,00	Referencia	
	Tradicional	2,43	(2,19–2,70)	< 0,001
	Moderno	2,21	(2,03–2,39)	< 0,001
Exposición				
	Sin amenorrea	1,00	Referencia	
	Con amenorrea	2,09	(1,92–2,27)	< 0,001
Mortalidad intrauterina y aborto espontáneo				
	No	1,00	Referencia	
	Sí	1,67	(1,53–1,80)	< 0,001
Indirectos				
Número ideal de hijos				
	≥ 5	1,00	Referencia	
	4	0,39	(0,32–0,48)	< 0,001
	3	0,16	(0,14–0,20)	< 0,001
	0–2	0,13	(0,11–0,15)	< 0,001
Edad (años)				
	15–19	1,00	Referencia	
	20–24	2,25	(1,92–2,63)	< 0,001
	25–29	5,49	(4,66–6,45)	< 0,001
	30–34	12,9	(10,7–15,5)	< 0,001
	35–39	27,3	(22,5–32,9)	< 0,001
	40–44	43,4	(35,7–52,8)	< 0,001
	45–49	61,3	(50,4–74,5)	< 0,001
Residencia				
	Urbana	1,00	Referencia	
	Rural	3,17	(2,88–3,50)	< 0,001
Trabajo				
	Independiente	1,00	Referencia	
	Para un familiar	0,69	(0,61–0,79)	< 0,001
	Para alguien más	0,31	(0,28–0,34)	< 0,001
Tipo de paga por el trabajo				
	No le pagan	1,00	Referencia	
	Solo dinero	0,37	(0,32–0,42)	< 0,001
	Dinero y especie	0,58	(0,48–0,70)	< 0,001
	Solo especie	0,74	(0,55–1,00)	0,052
Máximo nivel educativo				
	Sin educación	1,00	Referencia	
	Primaria	0,42	(0,33–0,54)	< 0,001
	Secundaria	0,08	(0,07–0,12)	< 0,001
	Superior	0,03	(0,02–0,04)	< 0,001
Quintiles de riqueza				
	Quintil inferior	1,00	Referencia	
	Segundo quintil	0,50	(0,45–0,56)	< 0,001
	Quintil intermedio	0,30	(0,27–0,34)	< 0,001
	Cuarto quintil	0,19	(0,17–0,22)	< 0,001
	Quintil superior	0,15	(0,13–0,17)	< 0,001

a Datos ponderados

En las mujeres con trabajo dependiente la posibilidad de tener hijos es menor que las que tienen una ocupación independiente. Esta desigualdad podría deberse al hecho de que las mujeres con un trabajo dependiente en ocasiones perciben la maternidad como un aumento del riesgo de desempleo y por ello posponen el embarazo (30, 31). En los trabajos dependientes, su ubicación respecto al hogar y la duración de la jornada laboral comportan una menor cantidad de tiempo disponible para el cuidado de los hijos, dos elementos que las mujeres tienen en cuenta al decidir procrear (32). Por otro lado, en estudios realizados en la Región de las Américas se sugiere que las políticas públicas insuficientes que no armonizan adecuadamente el trabajo y el papel de madre tienden a postergar la edad de la maternidad (20, 32). Las mujeres con aspiraciones de realización en su carrera profesional, en los trabajos dependientes, posponen la maternidad, ya que a las trabajadoras con hijos se les ofrecen ocupaciones de menor calificación o en ocasiones tienen menor probabilidad de ser contratadas (33). Del mismo modo, las mujeres jóvenes que se inician en el mercado del trabajo y que tienen bajos ingresos posponen el embarazo hasta que se han estabilizado profesionalmente y el salario percibido aumenta (2, 9, 20, 30, 31).

Con respecto a la incidencia del quintil de riqueza en la fertilidad, cuanto mayor riqueza menor es la fertilidad, algo ya notificado en la bibliografía (15, 26, 27). Es probable que las que gozan de mayores ingresos tengan un mayor nivel educativo y, por tanto, conocimientos sobre la planificación familiar y mayor acceso a los métodos de anticoncepción (9, 26). Posiblemente, esta relación inversa esté vinculada a su vez con un aumento del costo de oportunidad de la maternidad (26, 30, 32), que se exterioriza con una disminución salarial y menos oportunidades de formación (2, 9, 22).

Además, se ha observado que cuanto mayor es el nivel educativo menor es la fertilidad. Las encuestadas con educación secundaria y superior tienen niveles de fertilidad más bajos que las mujeres sin educación y estos resultados son similares a los publicados con anterioridad a esta encuesta (23–25). Las mujeres que tienen niveles educativos altos retrasan el momento de contraer matrimonio, que se considera como un periodo de exposición a la maternidad (3, 4, 15). Se sabe, a su vez, que los altos niveles educativos están asociados con el conocimiento y el aumento del uso de métodos anticonceptivos, cuya utilización frecuente se asocia con la reducción de los niveles de fertilidad (24–26). Cabe mencionar asimismo la relación que existe entre los niveles bajos de educación y los ingresos deficientes (16).

Cuanto mayor es el retraso del inicio de las relaciones sexuales, tanto menor es la fertilidad, pues se acorta el tiempo de la edad fértil (3, 4, 9). Una reducción similar de la fertilidad se observa con el aumento de edad en que se contrae el primer matrimonio debido a una reducción del periodo biológico fértil (3, 4, 9). También se sabe que la presencia de un cónyuge tiene un efecto positivo en la decisión de procrear (3). El matrimonio se percibe como una garantía de seguridad económica para criar a los hijos (16). Por otro lado, en las mujeres que tardan en casarse la posibilidad de tener niveles educativos altos y de acceder al mercado laboral es mayor (15).

**CUADRO 4.** Predictores de la disminución de los niveles de fertilidad de las mujeres peruanas que habían trabajado en la semana anterior a la encuesta (n = 13 848), Perú, 2014

Determinantes		Subgrupo de mujeres con ocupación laboral^a^		
OR crudo^b^		
OR	IC95%	P
Directos				
Edad en el primer matrimonio				
	10–18 años	2,34	(2,09–2,63)	< 0,001
	19–24 años	1,00	Referencia	
	25–46 años	0,39	(0,33–0,45)	< 0,001
Edad en la primera relación sexual				
	8–18 años	1,00	Referencia	
	19–24 años	0,38	(0,34–0,42)	< 0,001
	25–49 años	0,29	(0,24–0,34)	< 0,001
Edad al nacimiento del primer hijo				
	11–18 años	1,00	Referencia	
	19–24 años	0,41	(0,37–0,46)	< 0,001
	25–46 años	0,06	(0,04–0,07)	< 0,001
Estado civil				
	Nunca se casó	1,00	Referencia	
	Con pareja	42,1	(35,9–49,2)	< 0,001
	Otro	30,9	(25,8–37–0)	< 0,001
Uso actual de método anticonceptivo				
	Ningún método	1,00	Referencia	
	Tradicional	2,16	(1,91–2,45)	< 0,001
	Moderno	1,97	(1,78–2,18)	< 0,001
Exposición				
	Sin amenorrea	1,00	Referencia	
	Con amenorrea	2,30	(2,04–2,59)	< 0,001
Mortalidad intrauterina y aborto espontáneo				
	No	1,00	Referencia	
	Si	1,70	(1,53–1,88)	< 0,001
Indirectos				
Número ideal de hijos				
	5	1,00	Referencia	
	4	0,39	(0,30–0,50)	< 0,001
	3	0,17	(0,13–0,21)	< 0,001
	0-2	0,13	(0,12–0,17)	< 0,001
Edad				
	15–19	1,00	Referencia	
	20–24	1,87	(1,48–2,36)	< 0,001
	25–29	4,40	(3,47–5,57	< 0,001
	30–34	10,4	(8,11–13,4)	< 0,001
	35–39	22,6	(17,4–29,3)	< 0,001
	40–44	37,7	(29,1–48,75	< 0,001
	45–49	54,1	(41,0–70,1)	< 0,001
Residencia				
	Urbano	1,00	Referencia	
	Rural	3,63	(3,24–4,06)	< 0,001
Trabajo				
	Independiente	1,00	Referencia	
	Para un familiar	0,71	(0,62–0,81)	< 0,001
	Para alguien más	0,32	(0,29–0,35)	< 0,001
Tipo de paga por el trabajo				
	No le pagan	1,00	Referencia	
	Solo dinero	0,38	(0,33–0,44)	< 0,001
	Dinero y especie	0,64	(0,52–0,79)	< 0,001
	Solo especie	0,68	(0,49–0,95)	0,023
Máximo nivel educativo				
	Sin educación	1,00	Referencia	
	Primaria	0,47	(0,36–0,61)	< 0,001
	Secundaria	0,09	(0,07–0,12)	< 0,001
	Superior	0,03	(0,02–0,04)	< 0,001
Quintiles de riqueza				
	Quintil inferior	1,00	Referencia	
	Segundo quintil	0,46	(0,40–0,52)	< 0,001
	Quintil intermedio	0,27	(0,23–0,31)	< 0,001
	Cuarto quintil	0,17	(0,14–0,19)	< 0,001
	Quintil superior	0,12	(0,10–0,14)	< 0,001

a Mujeres con ocupación laboral en la semana anterior a la encuesta.

b Datos ponderados.

El mismo efecto reductor se relaciona con una valoración positiva de las familias pequeñas: en ellas el nivel de fertilidad y el número ideal de hijos se reducen. En la bibliografía se encuentran pruebas que respaldan el hecho de que el número ideal de hijos está influido por las normas sociales donde vive la mujer (19). En algunos sectores poblacionales de países de la Región se acumulan pruebas sobre una tendencia hacia el retraso de la maternidad debido a un debilitamiento del imperativo social de maternidad temprana (1, 34). Por otro lado, el número ideal de hijos es un factor importante en la disminución de la fertilidad, ya que, una vez alcanzada esta cifra, la maternidad se controla con la contracepción (15, 26). La discrepancia observada entre la fertilidad alcanzada y la fertilidad deseada, cuando esta última es mayor, constituye una tendencia actual (1, 2, 9, 12, 19) y esta diferencia podría ser el resultado de mayores niveles de incertidumbre e inestabilidad en la vida de las personas provocados por condiciones económicas y de trabajo adversas a nivel nacional y mundial (9, 20, 35). Estas situaciones desfavorables, conjuntamente con la desigualdad de género, afectan mayormente a las mujeres y a sus decisiones de procrear.

Tanto el ajuste de los modelos como su capacidad discriminante fueron aceptables, es decir, ambos modelos tienen buena capacidad discriminatoria de la fertilidad. Una explicación relativa se vincula con la elección de las variables predictoras a partir del modelo de Bongaarts (3, 4), que considera factores directos e indirectos, que impactan la fertilidad. Por otro lado, se debe tener en cuenta que en otra investigación publicada el ABC fue menor que las estimadas en el presente estudio (0,82) y que estas diferencias puedan deberse a la selección de las variables predictoras (24).

Las limitaciones de este estudio hacen referencia a la fuente de datos primarios utilizada (ENDES 2014), porque el objetivo principal de esta encuesta no es conocer la acción de los determinantes próximos en la disminución de la fertilidad, lo cual impide disponer de información de algunas variables. Por esta razón, no se pudieron estudiar algunos determinantes próximos, como, por ejemplo, el aborto inducido. La fuente de datos primarios no incluye información directamente procedente del cónyuge, si no de la mujer encuestada. Por consiguiente, no se pudo utilizar un enfoque que considerara todos los factores que permitirían explicar la complejidad del fenómeno de la reducción de la fertilidad.

Entre sus fortalezas cabe mencionar que la submuestra del estudio es grande y procede de una población exclusivamente formada por mujeres en edad fértil. A diferencia de otros estudios (12, 15, 23), se consideraron solo las mujeres con actividad sexual en las últimas cuatro semanas antes de realizar la encuesta independientemente del estado civil, ya que la fertilidad se produce también fuera de la vida en pareja.

En futuros estudios se recomienda realizar análisis comparativos a escala de la Región y por grupos de edad, para verificar la tendencia de los países hacia una discrepancia entre la fertilidad deseada y el número real de hijos, pues esto permitiría identificar patrones comunes y ayudar a interpretar el significado de tal diferencia. Asimismo, se recomienda realizar estudios que evalúen la efectividad de las políticas públicas, que, protegiendo la maternidad, promuevan una mayor fuerza laboral de las mujeres.

Existen diferencias entre los determinantes próximos (directos e indirectos) respecto a la disminución de la fertilidad, que se reflejan, en los factores directos, en un menor número de HNV entre las mujeres que iniciaron las relaciones sexuales, tuvieron su primer matrimonio y el nacimiento de su primer hijo después de los 25 años. Estos factores suponen un retraso de la maternidad en la población estudiada. Por otro lado, entre los determinantes indirectos, se puso de manifiesto un descenso de la fertilidad en las mujeres que tenían un número ideal de hijos menor o igual a dos, mayor nivel educativo y pertenecían al quintil superior de riqueza. Estos dos últimos elementos indican un nexo entre la disminución de la fertilidad y mayor autonomía económica de las mujeres. Además, el determinante trabajo puede tener un impacto en positivo en la disminución de la fertilidad con diferencias respecto a los tipos de ocupación de las mujeres. Cuanto mayor es la dependencia laboral de las mujeres menor es su fertilidad.

Sobre la base de estos resultados, se subraya la necesidad de poner en práctica políticas que fomenten la maternidad y el respeto de aquellas ya vigentes que regulan la licencia de maternidad y paternidad.

### Agradecimientos.

Los autores agradecen al Dr. Gian Battista Bolis sus útiles comentarios sobre el enfoque de la investigación y a los Dres. Nilda Montes Villanueva y Alexandre Ferraro, sus importantes observaciones de índole metodológica y estadística.

### Financiación.

El presente estudio ha sido autofinanciado.

### Declaración.

Las opiniones expresadas por los autores son de su exclusiva responsabilidad y no reflejan necesariamente los criterios ni la política de la *RPSP/PAJPH* o de la OPS.
